# Records of compass heading for long-distance ocean migrators show mid-ocean reorientation

**DOI:** 10.1126/sciadv.aed8113

**Published:** 2026-06-24

**Authors:** Graeme C. Hays, Kimberley L. Stokes, Giulia Cerritelli, Daniel P. Costa, Arina B. Favilla, Paolo Luschi, Matthew Rutishauser, Jared Tromp, Nicole Esteban

**Affiliations:** ^1^ Deakin Marine Research and Innovation Centre, School of Life and Environmental Sciences, Deakin University, Geelong, Victoria, Australia.; ^2^ Department of Biosciences, Swansea University, Swansea, UK.; ^3^ Department of Biology, University of Pisa, Pisa, Italy.; ^4^ Department of Ecology and Evolutionary Biology, University of California, Santa Cruz, Santa Cruz, CA 95060, USA.; ^5^ National Institute of Polar Research, 10-3 Midoricho, Tachikawa, Tokyo 190-0014, Japan.; ^6^ Wildlife Computers, 8345 154th Ave. NE, Redmond, WA 98052, USA.

## Abstract

The mechanisms by which animals navigate during long ocean migrations to specific targets remain equivocal despite over a century of investigation. To address this question, we developed and deployed a new tag that allowed the compass heading of migrators to be remotely relayed via satellite. On transocean migrations (>1000 kilometers and mean duration 27.5 days) between nesting and foraging sites, green turtles (*Chelonia mydas*) tended to perform sections of travel with a consistent compass heading, even if that led them off course, before reorienting. That migrating turtles did not continuously fine-tune their heading but rather made occasional reorientations is consistent with the suggestion that they use geomagnetic signposts, or other crude maps, to facilitate occasional course corrections.

## Teaser

Records of compass headings show that migrating green turtles only occasionally reorient during ocean crossings.

## INTRODUCTION

How animals navigate during long-distance migrations to specific targets has been an awe-inspiring mystery for a century or more (*1*–*2*). During this time, many advances have been made in understanding how these navigational challenges are overcome. For example, it is known that many animals can maintain a particular compass heading by using the sun or other celestial compass [e.g., (*3*–*4*)]. However, in addition to a compass sense, animals also need a mechanism to estimate their position with respect to the goal, for example, through a map sense or the use of cues emanating from the goal itself. In the oceans far from land, navigational challenges may be acute because of the absence of physical landmarks and the consequent need for alternative navigational information such as the use of Earth’s geomagnetic field (*5*). Geomagnetic maps may help guide long-distance ocean migrations. For example, laboratory studies have shown that sea turtles can sense components of the Earth’s geomagnetic field, specifically inclination and intensity, which may form a bicoordinate magnetic map in the oceans (*6*). Further evidence for this use of geomagnetic maps has come from observations of turtle disorientation in the open ocean when equipped with magnets (*7*–*8*). The combination of long migrations to specific targets along with seminal work on magnetic orientation has made sea turtles an iconic group for studies of long-distance migration and navigation.

A further complication for migrating ocean swimmers is that ocean currents may deflect animals from their intended course, with an animal’s movement being the sum of its active swimming and advection by ocean currents (*9*–*10*). The same deflection may also occur with flying birds and insects due to the wind (*3*). Partitioning these two components of an animal’s movement is not straightforward and, over large scales, has typically involved assessing the course over the ground by satellite tracking and the ocean current vector from ocean models (*11*–*13*). This approach is limited by the availability of global ocean current models, which are relatively coarse and only approximate the ocean currents migrants experience (*14*). In theory, measurement of an animal’s compass heading could be used to assess whether course changes in migration are the result of a change in heading or simply due to a change in the ocean current. However, compass heading measurements have only been available from data loggers that have to be retrieved for data recovery [e.g., (*15*–*16*)], which has precluded their use for animals migrating huge distances that are unlikely to be resighted.

Here, we describe the development and deployment of a compass data logger that relays compass headings along with animal locations for many months from remote areas and over extended migrations. We deployed this tag on sea turtles to test whether changes in the ground track during migration were driven by changes in an animal’s compass heading, indicative of reorientation decisions, or whether changes in the direction of the ground track were simply the passive consequence of changing ocean currents. In this way, we addressed whether migrating sea turtles show occasional reorientation or continually fine-tune their heading.

## RESULTS

**Fig. 1.**
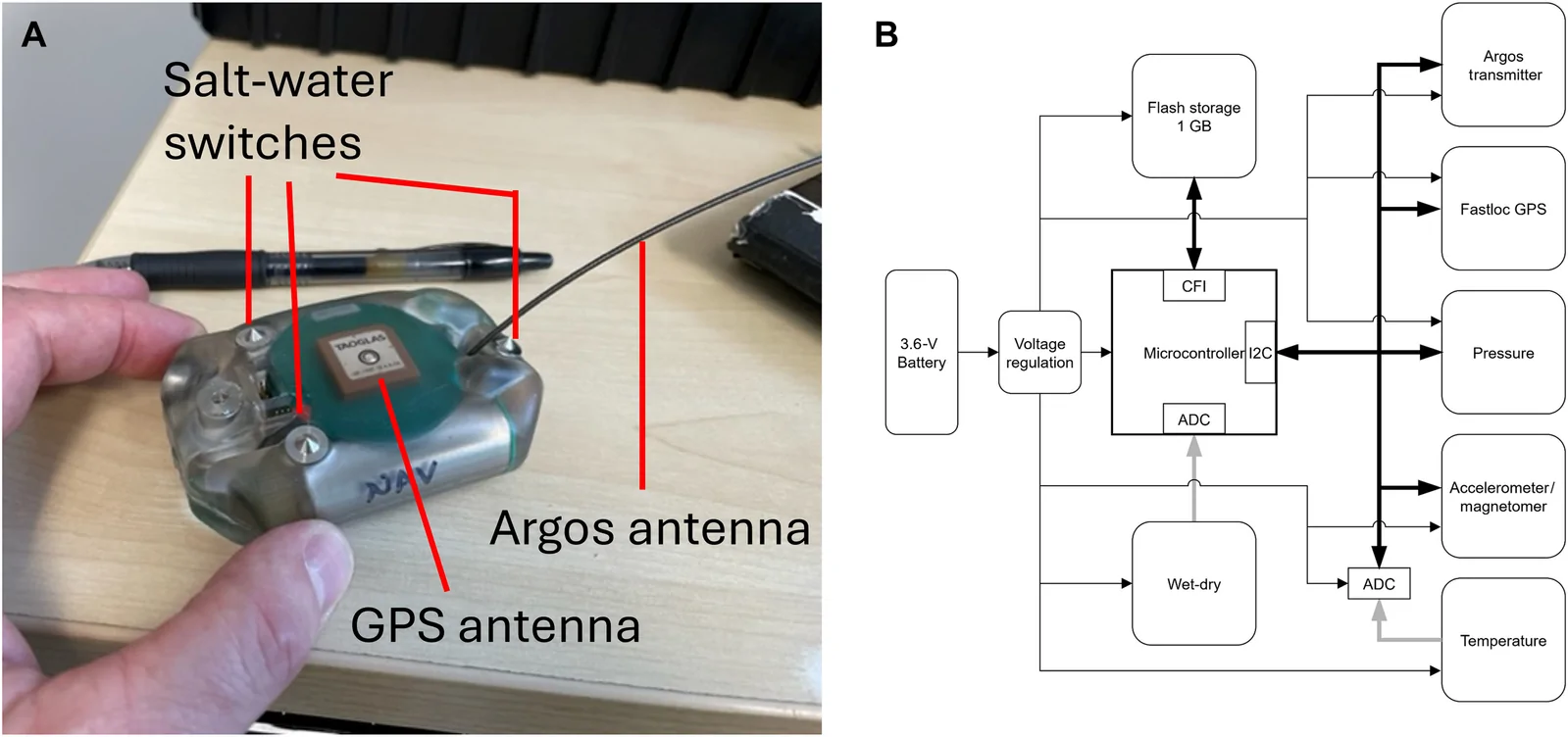
The layout of the SCOUT-NAV-409 tag. (**A**) Finished tag showing GPS antenna, Argos antenna, and salt-water switches (wet-dry sensors). Photo credit: M. Rutishauser. (**B**) Hardware architecture. Thick black connections indicate digital signals, gray connections indicate analog signals, and thin lines indicate power connections. ADC, analog-to-digital converter; CFI, common flash memory interface; I2C, inter-integrated circuit. The salt-water switches shown in (A) are used to determine whether the tag is wet or dry, i.e., the wet-dry state shown in (B).

**Fig. 2.**
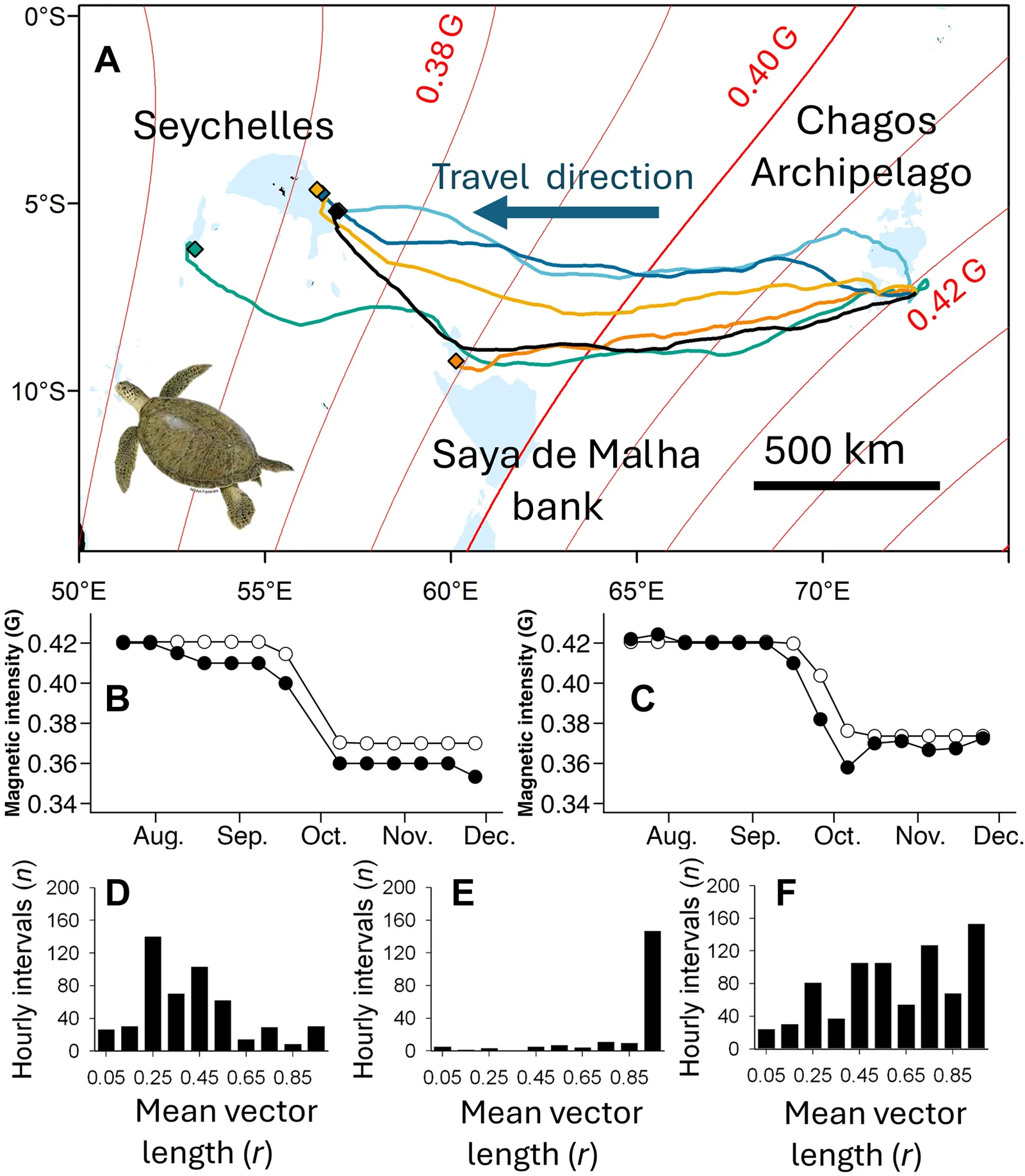
A summary of the tracks and heading data for migrating green turtles. (**A**) The tracks of six individuals from the Chagos Archipelago to their foraging grounds in the Seychelles and on the Saya de Malha bank. Red lines show magnetic field intensity (10,000 G = 1 tesla). Light blue areas show submerged banks (<500 m). Turtle image courtesy of National Oceanic and Atmospheric Administration (NOAA). (**B** and **C**) Two examples of the magnetic field intensity relayed from tags (filled symbols) and the magnetic field intensity mapped at that location by NOAA (open symbols). As turtles migrated westward, the decline in the magnetic field intensity is evident. (**D** to **F**) Frequency distributions of the mean vector length for the 1-s compass headings within each hour during the nesting season (D), during migration (E), and on the foraging grounds (F). A high mean vector length (close to one) indicates a very consistent compass heading within that hour. Mean vector length data are shown for one individual (turtle ID #265009), but these patterns were consistent across all individuals (fig. S3).

In the field at a mid-ocean nesting ground (Chagos Archipelago, Indian Ocean), we instrumented six nesting female green turtles (*Chelonia mydas*) with tags that provided extensive compass heading information, using a compass sensor interfaced with a satellite transmitter (Fig. 1). The compass sensor measured the heading of the turtle directly with respect to true north using a magnetometer. The turtle heading measured directly in this way may be different from the turtle’s direction of travel due to the impact of ocean currents, which can be measured from the track. After the nesting season in the Chagos Archipelago, the six equipped turtles all migrated westward, arriving at their foraging grounds in the Seychelles or on the Saya de Malha bank (Fig. 2A). Across these six individuals, migration took on average 27.5 days to complete (range = 19.8 to 33.7 days) and the mean number of hours for which compass data were received during migration was 171 hours (range = 112 to 236 hours), reflecting a mean of 27.7% (range = 13.9 to 49.7%) of each migration track.

As the turtles headed westward on their migration, the magnetic field intensity relayed via the tags declined in line with known magnetic field intensity at the successive turtle locations (Fig. 2A). Immediately before, during, and after migration, the mean difference between the measured and actual magnetic field intensity averaged 0.015 G (SD = 0.01 G), i.e., any sensor drift was expected to produce a resulting compass heading error of <<10° (Fig. 2, B and C, and fig. S2).

Between the time spent in the nesting season, during migration and on the foraging grounds, there were significant differences in the compass heading mean vector length for the 1-Hz measurements within each hour (Fig. 2, D to F, and fig. S3). The mean vector length of each individual during migration (mean= 0.91, SD = 0.04, *n* = 6 individuals) was significantly higher than during either the nesting season (mean 0.46, SD = 0.09, *n* = 6 individuals) or on the foraging grounds (mean = 0.57, SD = 0.05, *n* = 6 individuals) (*t* ≥ 10.5, *P* < 0.001). These results indicate that across all individuals during the migration, the turtles generally moved in a very consistent direction within each hour (mean vector length values were generally high and close to one, indicating low variance between 1-s compass measurements) (Fig. 2E and fig. S3). In contrast, during the nesting season and while on the foraging grounds, the turtles did not have a consistent direction of travel (low mean vector length values indicating high variance between 1-s compass measurements within each hour) (Fig. 2, D and F). During migration, the mean vector length was generally high during both day and night with no consistent diel pattern (fig. S4, *t* = 1.56, *P* = 0.18).

**Fig. 3.**
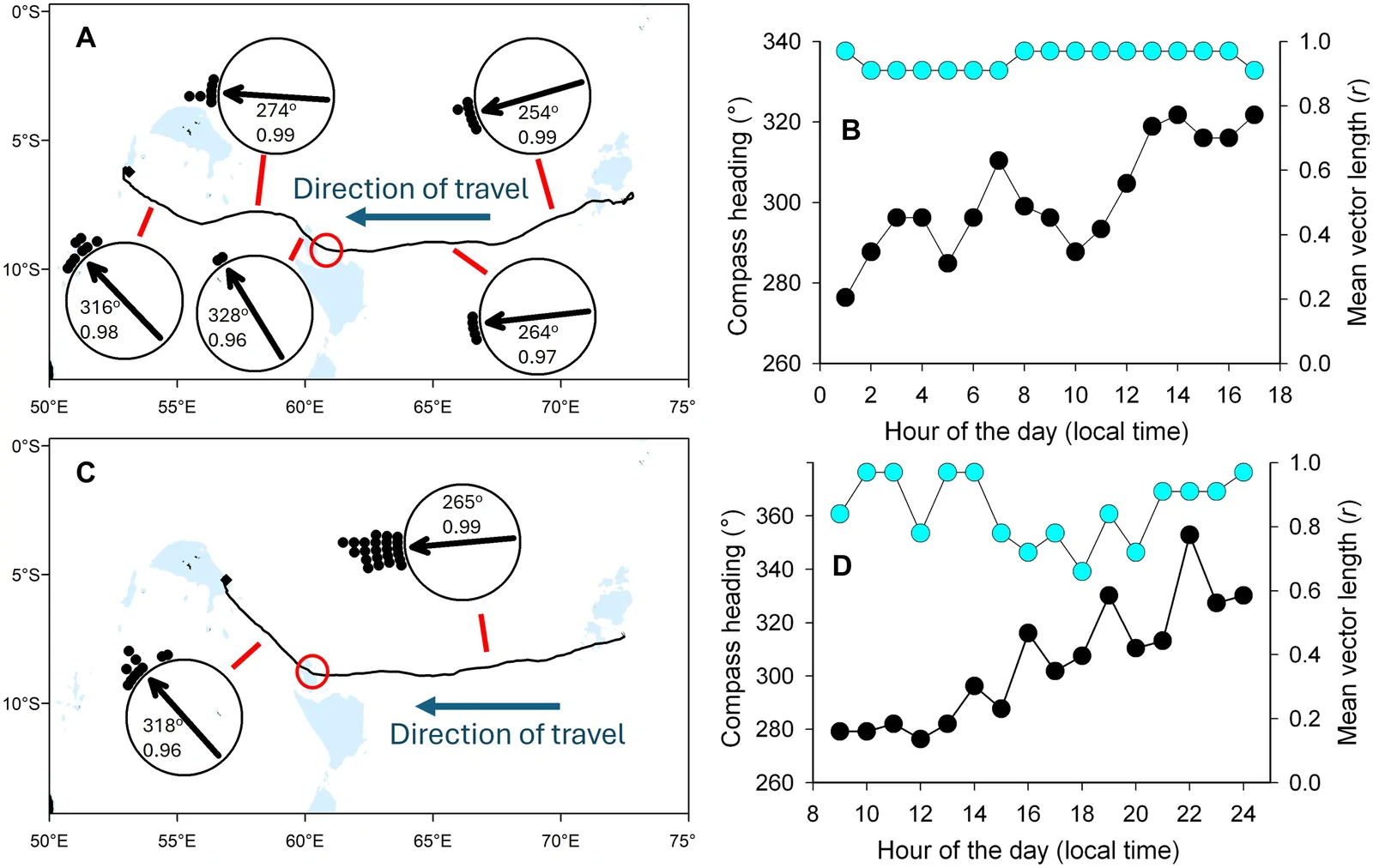
Examples of migration tracks and associated compass heading information for postnesting female green turtles migrating from the Chagos Archipelago to the Seychelles. (**A** and **C**) Each circular plot shows a summary of the compass headings relayed from the tag for a section of the track, illustrating how the compass heading changed during migration from one track section to the next. For clarity of the plot, each point in the circular plots represents 5 hours of data in that 5° heading bin, although all the single 1-hour datasets underlying these plots were used for the calculations. Most values for the mean vector length were close to 1 (see Fig. 2E and fig. S3), indicative of a well-orientated heading throughout that hour. The arrows and numbers inside the plots indicate the mean vector orientation and length for that segment. Turtle IDs #265002 [(A) and (B)] and #265009 [(C) and (D)]. The red circle in each plot indicates the location of the data expanded in (B) and (D). (**B** and **D**) Individual mean hourly compass heading measurements (black points), relayed during migration, indicate a switch in compass heading on 10 September and 2 October for these two turtles, respectively, corresponding to turns in their tracks. Blue points, the mean vector length for each hour, indicate well-orientated heading throughout that hour.

There were clear changes in the compass heading along the tracks (Fig. 3). We divided each track into sections where the turtle travel direction, calculated from the Fastloc GPS locations, was both consistent (straightness index >0.97) and well sampled [compass heading data were obtained for a mean of 66% (range 22.2 to 100%, SD = 24.9%) of the hours in track sections]. Across individuals, a total of 26 track sections were examined (mean straightness index = 0.986, SD = 0.025, *n* = 26). For example, for turtle #265002, when circular plots were examined of the headings recorded within each track section, it was evident that the mean compass heading showed distinct changes between different parts of the migration track (Fig. 3A). For example, in the early parts of this migration, there was often a WSW compass heading, while in latter stages, there was often an NW compass heading (Fig. 3A). For turtle #265009, there was a distinct change in compass heading on 2 October, with a switch from a generally SW heading to a more NW heading (Fig. 3C). The differences in compass headings between successive track sections for these two turtles were always significant (Watson’s two-sample U^2^ comparing the 1-hour mean heading values recorded between successive track sections, *P* < 0.05). The change in compass heading associated with turns in the ground track could take many hours to complete, i.e., individuals did not instantaneously switch to a new compass heading. The mean 1-hourly data from these gradual turns are shown for both turtles #265002 (Fig. 3B) and #265009 (Fig. 3D), with the compass heading changing gradually over about 15 hours. We examined 26 track sections across six tracks corresponding to 20 reorientations between sections. Only one of these 20 reorientations was initiated in water shallower than 500 m, occurring on the Saya de Malha bank, while the rest were in very deep (1000+ m) oceanic water.

**Fig. 4.**
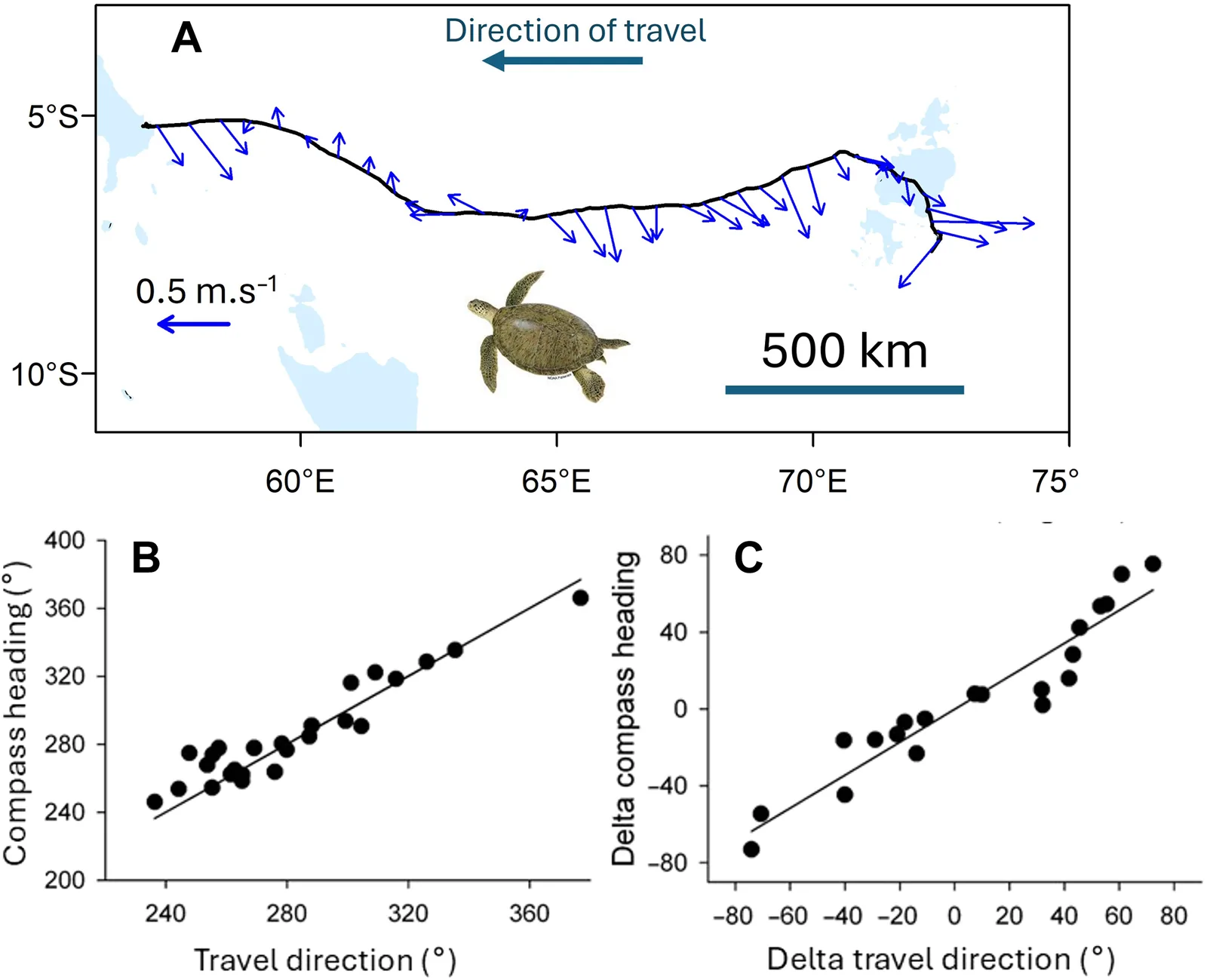
Links between compass heading, direction of travel, and local ocean currents. (**A**) An example of the direction and magnitude of the ocean currents along a migration track (turtle ID #265006). While the ocean current was determined for 1-hour intervals, plotted for visual clarity are the mean current direction and speed per 24 hours. Turtle image courtesy of NOAA. (**B**) For track sections from all turtles the relationship between the travel direction (from first and last Fastloc GPS points for that track section) and the mean compass heading for that track section (*F*_1,24_ = 219.4, *R^2^* = 0.90, *P* < 0.001). (**C**) For track sections from all turtles, the change in travel direction between successive sections of the track versus the change in the mean compass heading between those successive sections (*F*_1,18_ = 169.0, *R*^2^ = 0.90, *P* < 0.001).

The mean travel speed of turtles on track sections was 0.60 m s^−1^ (SD = 0.25 m s^−1^, *n* = 26) versus a mean ocean current speed of 0.29 m s^−1^ (SD = 0.18). For each track section, we determined the mean compass heading from all the individual 1-hour mean compass heading values (e.g., Fig. 2, A and C) as well as the mean current direction. Further, the direction of travel was calculated between the first and last Fastloc GPS points for that track section. Across track sections, the mean modular difference between the turtle heading and the current direction was 67° (SD = 48°, range: 3° to 164°) (Fig. 4A and fig. S5). The mean compass heading for track sections was strongly correlated with the travel direction (Fig. 4B). While the compass heading and the direction of travel were very similar here, implying that ocean currents did not advect migrating turtles laterally in any major way, in other cases such as with weaker swimmers or stronger currents, an individual’s compass heading might differ to its direction of travel. When we analyzed the travel direction for each section of track using stepwise multiple regression with the corresponding mean compass heading, mean current direction, mean current speed, and U and V vectors (eastward and northward components, respectively), we found that compass heading explained 90% of the variance in travel direction, with none of the residual variation being explained by the ocean currents. The resulting equation after forward selection was travel direction = 1.05 (compass heading) + 1.05 (*F*_1,24_ = 219.4, *R*^2^ = 0.90, *P* < 0.001).

When the change in travel direction between successive track sections was analyzed using stepwise multiple regression with the change in the mean compass heading and the change in mean ocean current properties (intensity, direction, and U and V components), the change in compass heading explained 90% of the change in travel direction: change in travel direction = 1.05 (compass heading) + 0.78 (*F*_1,18_ = 169.0, *R*^2^ = 0.90, *P* < 0.001) (Fig. 4C). Only 3.9% of the residual variation was explained by the current properties (specifically the change in current direction). There was no significant difference between the change in travel direction and the change in mean compass heading between successive sections (paired *t*-test, *t* = 0.35, *P* = 0.73, *n* = 20), i.e., the magnitude of the change in travel direction was very similar to the magnitude of the change in mean compass heading (mean difference = 1.09°, SD = 13.9°). Together, these results indicate that almost all changes in travel direction were due to the turtles' change in mean compass heading.

## DISCUSSION

The compass tags provide a step advance in animal orientation studies by allowing the interaction between animal heading and travel heading to be directly explored across a range of taxa traveling long distances across the open ocean and in a range of current regimes. We have demonstrated through direct measurements that migrating marine turtles reorientate in the open ocean and that their courses are driven primarily by their compass heading rather than ocean currents. These findings from free-living individuals help complete the picture of the processes that underpin target finding during long-distance ocean migrations. From many satellite tracking studies, it is clear that sea turtles do not complete long open-ocean migrations to remote targets with pinpoint precision (*17*–*18*). Rather, individuals veer off direct routes to their targets, they may overshoot their targets, sometimes by hundreds of kilometers, and they often perform circuitous search patterns in the final stages of migration (*17*). Our compass-heading information suggests that turtles can only approximate their location in the open ocean, indicative of a crude map sense, as also hypothesized from laboratory evidence of their sensory abilities (*5*). While this crude map sense might be based on geomagnetic inclination and intensity (*5*), it could equally be based on other, as yet unidentified, information. As more data emerge about where turtles reorientate on their migrations, it might be possible to identify whether there are specific signposts, e.g., magnetic, which initiate heading changes or alternatively if heading changes occur when individuals are sufficiently off-course that they can detect that they need to turn. There might be alternative explanations for nondirect travel during sea turtle migration such as travel to intermediate refueling targets, as sometimes occurs in birds. However, green turtles do not stop en route to their final destination, suggesting that refueling does not occur to any significant extent.

The degree to which cross flows (e.g., ocean currents or winds) affect an animal’s travel direction depends on the relative magnitudes of the animal’s active movement and the environmental flows. For example, the travel direction of migrating insects can be influenced by wind direction to the extent that insects time their travel to favorable wind directions (*19*). Recent evidence from direct tracking of migrating insects shows that they can also adjust for head and crosswinds to precisely hold course (*20*). Even for strong flyers such as large birds, wind patterns may be important (*21*). For example, ospreys migrating across Africa may die after being carried by storms out into the Atlantic (*22*). Set against this backdrop, our results showed that for green turtles migrating across the Indian Ocean, ocean currents play a much smaller role in driving the animal’s travel direction than active swimming. The swim speed of a migrating turtle is expected to be faster than ocean currents (*9*). However, there might be situations where strong crosscurrents affect the travel heading of even powerful swimmers like adult turtles, and currents are also expected to play a larger role in driving the movements of pelagic juvenile sea turtles (*23*).

Data loggers that are recovered from animals for data download can measure across many channels at fast sampling intervals and have allowed huge advances in understanding the behavioral and physiological ecology of many species (*24*, *25*). The tag we described here with a compass sensor interfaced to a satellite transmitter, enables remote data relay, eliminating the need to recover datalogging compass tags. This breakthrough enables compass orientation studies in a range of long-distance ocean migrants, including marine mammals and pelagic fish (*26*, *27*). A key to successfully capturing compass heading data in remote locations was the minimal drift in the compass sensor, even for deployments lasting many months. Further, planned increases in the number of satellites within the Argos tracking system (*28*) will increase the bandwidth for daily data transmission enabling more compass data to be relayed remotely. As more turtles are tracked with compass sensors and as Argos bandwidth increases, there will be fewer gaps in the compass data during migration, making it possible to identify patterns in where reorientation events occur. Furthermore, using breakpoint analysis will improve understanding of what drives these events and where they occur.

We have demonstrated that sea turtles can maintain consistent compass headings for extended periods during migration, both within individual hours and across hours and days. This consistency in compass heading during both daytime and nighttime is noteworthy. Sea turtles have eyes adapted for underwater vision and are myopic in air (*29*). Therefore, in contrast to some birds that migrate at night (*30*–*31*), sea turtles likely cannot clearly see celestial cues such as stars. Hence, the consistent day-night headings we recorded are best explained by the turtles’ reliance on a magnetic compass, which would function at night and at depth, where access to celestial cues is not possible. It cannot, however, be excluded that turtles may additionally use celestial compasses in some conditions, such as a sun compass during the day, which is widely used by migrating taxa (*32*). The consistent day and night compass headings also suggest that migrating sea turtles do not sleep or rest for long periods at night, which contrasts with their nocturnal resting at their breeding and foraging areas, where they sleep on the seabed (*33*). Some migrators, including many bird species, can travel long distances across many weeks without much rest (*34*–*35*). The specific physiological mechanisms that might allow sea turtles to endure extended periods without much rest are unknown (*36*).

When a consistent compass heading leads a turtle off the direct route to the goal, additional orientation mechanism(s) may then enter into play. For instance, as a turtle travels off course, the local geomagnetic field (inclination and intensity) will increasingly deviate from that along the straight route to the target. Consequently, even with a crude geomagnetic map sense, as a turtle travels farther from the direct route to its target, it will be increasingly likely to detect that it is off course and so make a compensatory reorientation. Using this navigation process, it is expected that individuals will never travel endlessly further-and-further off-route without redirecting their path goalward, a pattern seen widely in sea turtle tracking data (*17*–*18*). This navigation strategy likely gets turtles close to their destination, with the final targeting facilitated by other processes such as local search, wind-borne cues, sea-bed topography, or landmarks in the case of coastal targets (*6*). Similarly, other animals may have a hierarchy of navigational mechanisms to arrive at their goals, which can include local search (*2*).

When turtles reorient in the open ocean, the compass heading can gradually change over 24 hours, i.e., reorientation turns did not happen instantaneously. This pattern of gradual change contrasts to that seen when turtles encounter a mainland coast where they face a simple binary choice, turn left or right, and so quickly reorientate (*16*). Similarly, animals migrating over land, including birds, may show rapid reorientation in response to landmarks (*37*). Evidence that turtles take several hours to settle on a new compass heading during mid-ocean turns suggests the use of a crude map sense as it takes an individual some time to perceive how its new heading affects its movement within the map it perceives.

While some migrating animals may have systems for constantly fine-tuning their course to compass heading [e.g., (*20*)], our evidence from the compass heading of individuals suggests that sea turtles, iconic long-distance migrators, complete open-ocean migrations to small targets with only an approximate map sense so that they often head off the beeline to the target. This navigational system might be described as “not perfect, but adequate.” Turtles are known to have tight fidelity to particular nesting and foraging areas, with individuals returning to the same locations after breeding migrations (*38*). Our findings show they often complete these journeys via circuitous routes with only occasional course corrections in the open ocean.

## MATERIALS AND METHODS

### Tag design

The SCOUT-NAV tag (SCOUT-NAV-F-409A) was designed to provide compass heading for free-ranging turtles during their migration. The primary design requirements were the following: (i) measure compass heading to within 10°, (ii) determine location to within 100 m, (iii) obtain the data without physically recovering the tag, and (iv) operate for at least 6 months. Tag size and pressure tolerance also constrained the design. Additional considerations included the need for tilt compensation of the compass heading as we could not assume that the tag was horizontal, and the ability to transmit data without relying on land-based infrastructure on an animal with brief and limited time at the surface. Figure 1 shows the finished tag and the system architecture.

### Compass heading

Compass heading was derived from the accelerometer and magnetometer calibrated using a least squares ellipsoid technique (*39*) as implemented in Magneto (*40*). The calibration data were gathered by tumbling the tag for roughly 1 min, exploring all orientations. The calibration was then checked with a second tumble for offset and distortions against a sphere centered at the origin of a Cartesian frame. Acceleration and magnetic field were measured on three axes (*x*, *y*, and *z*). The magnetic field was measured and logged at 1 Hz. Acceleration was measured at 25 Hz for tilt compensation and logged at 1 Hz. Depth and temperature were recorded at 1 Hz to assess animal behavior as well as for temperature and pressure compensation of the magnetometer and accelerometer. Compass heading for Argos relay was summarized on-board the tag as the average compass heading over 1 hour along with the mean vector length (*r*) (*41*), a measure of the dispersion of the heading data, which varied from 0 to 1 with values close to 1 indicating that the headings were very similar (i.e., little dispersion and high directionality) and values closer to 0 indicating a broad dispersion of headings (i.e., high dispersion and no directionality of animal movement). To assess sensor drift, the magnetic field was averaged each hour on-board the tag and relayed via Argos for comparison against published charts of field intensity (see below).

### Telemetry and location

Integrating an Argos transmitter into the tag allowed remote data transmission. Argos data can be sent without a handshake or acknowledgment of receipt, allowing for data transmission when the tag is at the surface and the satellite is in view for one second or less. While the Argos satellite system can provide locations, the accuracy is relatively low; we therefore incorporated Fastloc GPS to provide more accurate locations (*42*). Following procedures used previously, only Fastloc GPS positions obtained with a minimum of four satellites and a residual error value of less than 35 were used, producing locations that were generally within a few tens of meters of the true location (*42*).

### Field deployments

SCOUT-NAV tags were attached to the carapace of nesting adult female green turtles on the island of Diego Garcia (7.428°S, 72.458°E) in the Chagos Archipelago using quick-setting epoxy (*43*) taking care to position the tag along the animal’s midline such that the Argos antenna was pointing directly forward. The accelerometer and magnetometer of each tag were calibrated just before deployment, as described above. Of the eight tags attached, six provided extensive compass heading information during migration, which we examine here. Although the other two tags provided some data, the lack of extensive data from these remaining two tags likely reflects limited time at the surface and, hence, limited Argos data transfer. Tags relayed compass heading data with respect to magnetic north, and these values were corrected during data processing for declination (https://ngdc.noaa.gov/geomag/calculators/magcalc.shtml#igrfgrid) to produce compass headings with respect to true north.

### Tag data analysis

We examined several metrics, such as the mean vector length, throughout migration, with the specific goal of establishing whether changes in travel direction reflected shifts in compass heading. We then compared the turtle’s travel direction with its mean compass heading, calculated for the corresponding sections of the track. Differences between these two headings are expected because of ocean currents since a turtle’s track over the ground is the sum of its active swimming and ocean currents.

### Ocean currents

U (east-west) and V (north-south) components of the ocean currents synoptic with the turtle locations were retrieved from 1-hour Copernicus Global Ocean Physics Analysis and Forecast models, which has a spatial resolution of 0.08° (about 8.9 km at these latitudes) (https://doi.org/10.48670/moi-00016). The sea current flows are modeled at varying depths and we chose to use the data from a depth of 0.5 m, given that migrating turtles generally remain in the upper layers of the water column (*44*).

### Magnetic field intensity maps

Maps of magnetic field intensity in the region were obtained from the National Oceanic and Atmospheric Administration National Center for Environmental Information (https://ngdc.noaa.gov/geomag/calculators/magcalc.shtml) and used to assess tag sensor drift.

### Statistical analyses

Statistical analyses, including stepwise multiple regression, were conducted using Minitab ver. 8.2 Extended and R (*45*).

### Ethics statement

The study was approved by Swansea University and Deakin University Ethics Committees (approval numbers IP-2122-17 and AEX09-2017, respectively) and the British Indian Ocean Territory (BIOT) Administration of the UK Foreign, Commonwealth, and Development Office. The study was endorsed through research permit 0007SE24 from the Commissioner’s Representative for BIOT, and the research complied with all relevant local and national legislation.
