# Supplementary material for: Records of compass heading for long-distance ocean migrators show mid-ocean reorientation
Source: Sci Adv. 2026 Jun 24;12(26):eaed8113. doi: 10.1126/sciadv.aed8113 (PMC13292924; doi:10.1126/sciadv.aed8113)
Supplement: Supplementary file 1 — Supplementary Text Figs. S1 to S5 Legends for tables S1 and S2 [file sciadv.aed8113_sm.pdf]

Science Advances
AAAS

Supplementary Materials for

Records of compass heading for long-distance ocean migrators show mid-ocean reorientation

Graeme C. Hays *et al.*

Corresponding author: Graeme C. Hays, g.hays@deakin.edu.au

Sci. Adv. **12**, eaed8113 (2026)
DOI: 10.1126/sciadv.aed8113

This PDF file includes:

Supplementary Text
Figs. S1 to S5
Legends for tables S1 and S2

Other Supplementary Material for this manuscript includes the following:

Tables S1 and S2

Supplementary Text

Tag design verification: compass heading and long-term stability

To demonstrate that the system could meet the 10° accuracy requirement for the mean heading recorded per hour, tags were compared against a handheld compass while walking with the tags at various headings and tilts. The error in the measured compass heading was always < 5°. To examine the impact of potential drift in magnetometer readings that may occur during field deployments, several trials were conducted in which an offset was intentionally added to the magnetometer. An equal offset of 0.04 G was added to each magnetometer channel resulting in a magnetic field intensity with an error of 0.07 G. The resulting compass heading error ranged from 2 to 14° averaging 9.1°, i.e. with sensor drift resulting in a magnetic field intensity error of <0.07 G, the remotely relayed compass heading values would be accurate to within 10° on average (Fig. S1). Note 10,000 Gauss = 1 Tesla. Further, a tag was deployed on land (not attached to an animal) for over 80 days with interruptions to download and check for sensor drift. The maximum deviation on any magnetometer channel was 0.02 G, which suggests the 0.04 G offset chosen for our trials provides a conservative estimate of the compass heading's accuracy, i.e. there was low sensor drift over time.

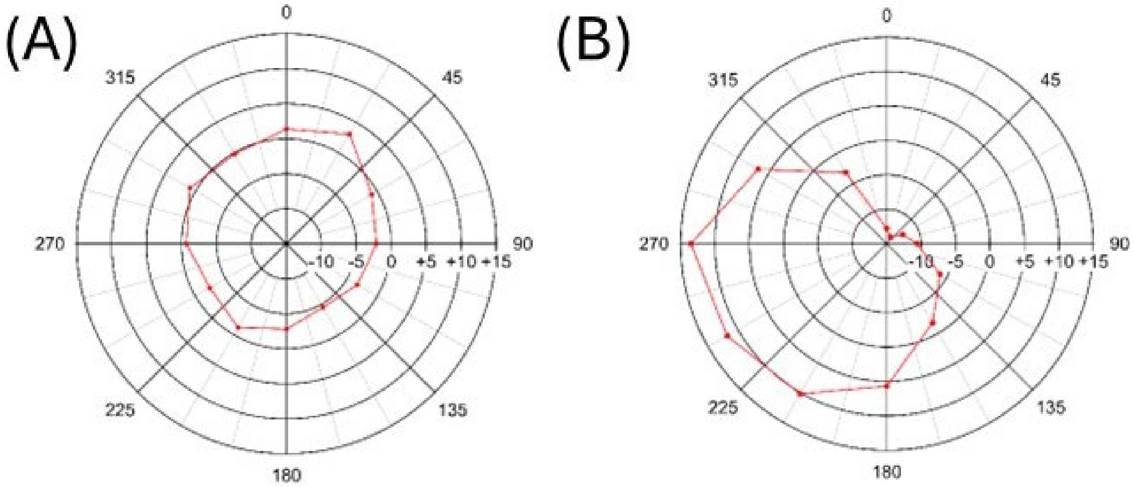

Fig. S1. Heading errors and simulated drift of the compass sensor. (A) The typical heading error after calibration was 0-5 degrees across all compass bearings. Heading error scale is indicated on the horizontal axis. (B) After intentionally adding an offset so the error in the magnetic field intensity was 0.07 G, the resulting compass heading error ranged from 2 to 14 degrees across all compass bearings. Note 10,000 Gauss = 1 Tesla.

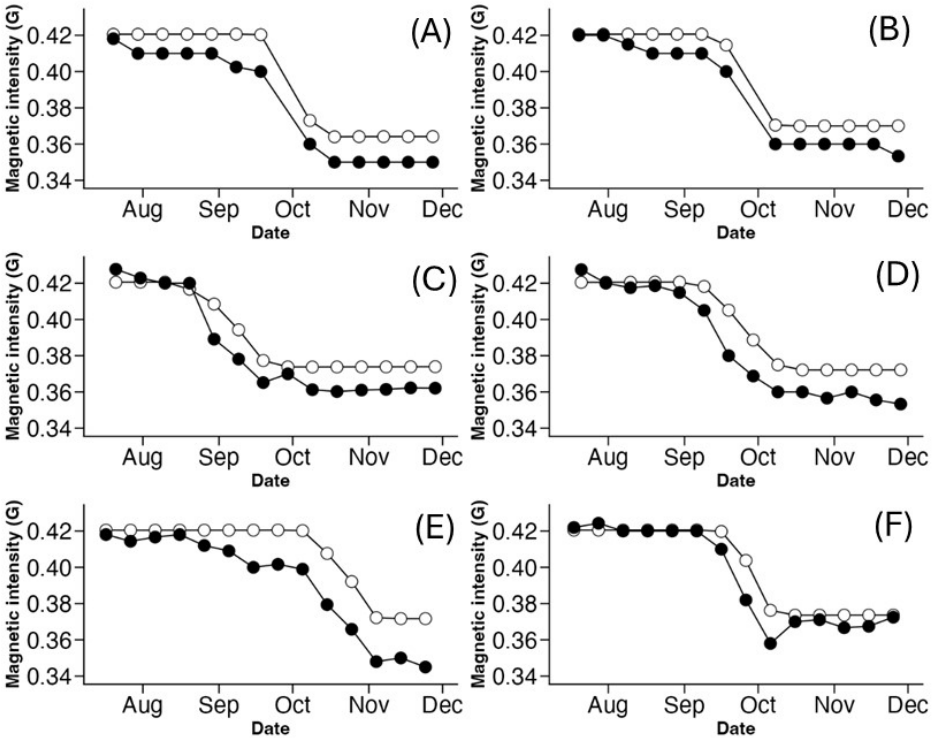

Fig. S2. Compass drift across months of tracking. Across all tags the magnetic field intensity relayed from tags (filled symbols) and the magnetic field intensity mapped at that location by NOAA (open symbols). Panels A-F are turtle IDs #265002, #265004, #265006, #265007, 265008 and #265009. Data from tags #265004 and #265009 are also shown in Fig. 2 as examples. The period when the magnetic intensity declined corresponds with when each turtle was migrating. Note 10,000 Gauss = 1 Tesla.

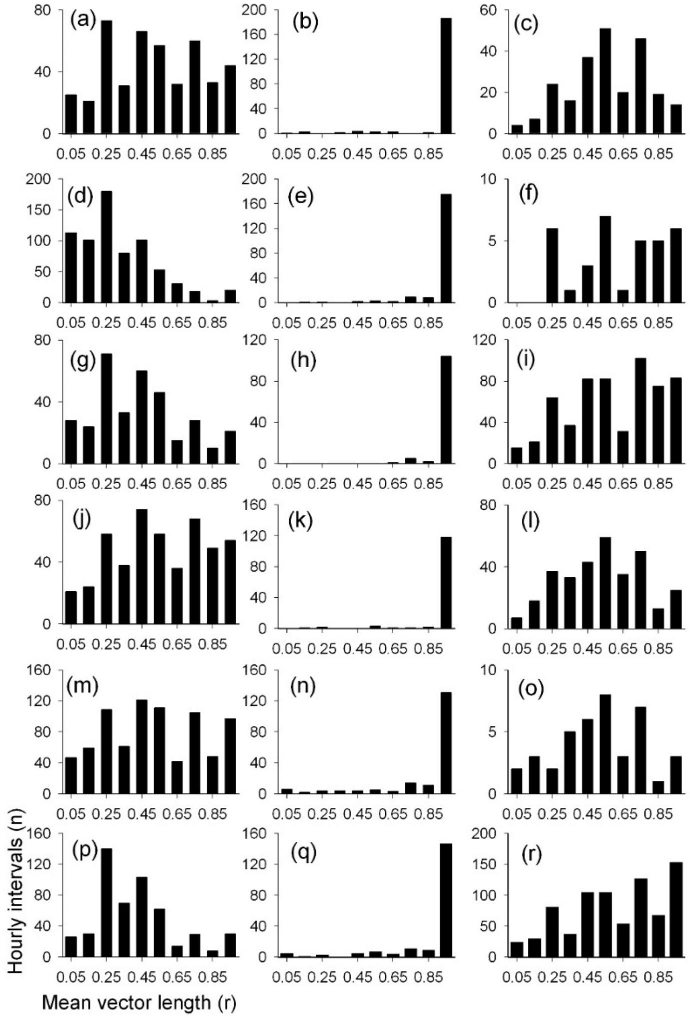

Fig. S3. Variation in directed swimming across migratory phases. Frequency distributions of the mean vector length for the 1-sec compass headings within each hour during the nesting season (left panels), during migration (center panels) and on the foraging grounds (right panels) for six tags. A high mean vector length (close to one) indicates a very consistent compass heading within that hour. Turtle IDs (A-C) #265002, (D-F) #265004, (G-I) #265006, (J-L) #265007, (M-O) #265008, (P-R) #265009.

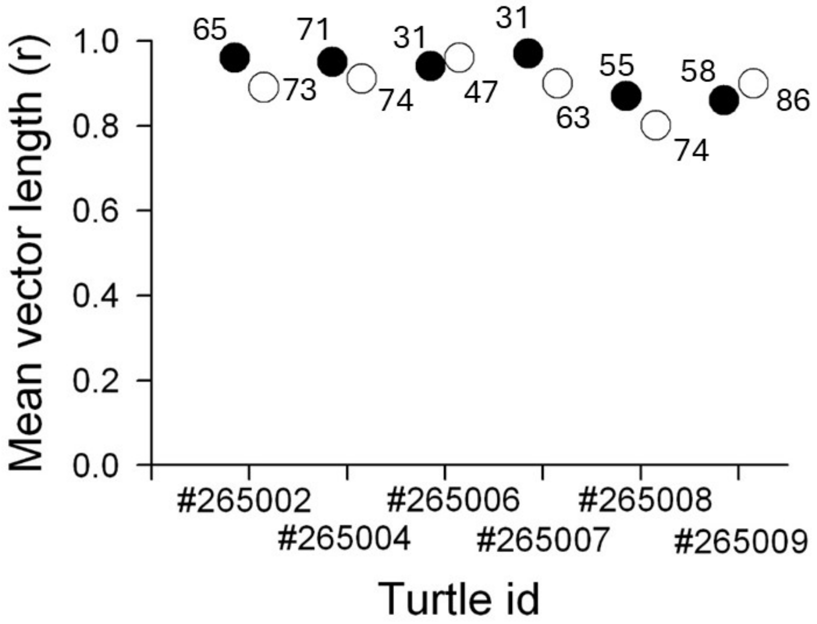

Fig. S4. Diel patterns in course consistency. The mean vector length (a measure of the consistency of compass headings within each hour) for night (filled circles) and the day (open circles) during migration for six individuals. SE bars are smaller than the plot symbols. Night and day were defined as local midnight +/- 4 hours and local midday +/- 4 hours respectively. The sample size (number of hours of data) is indicated next to each point. Across these six individuals, there was no significant difference between the mean vector length during the day versus the night ($t = 1.56$, $P = 0.18$).

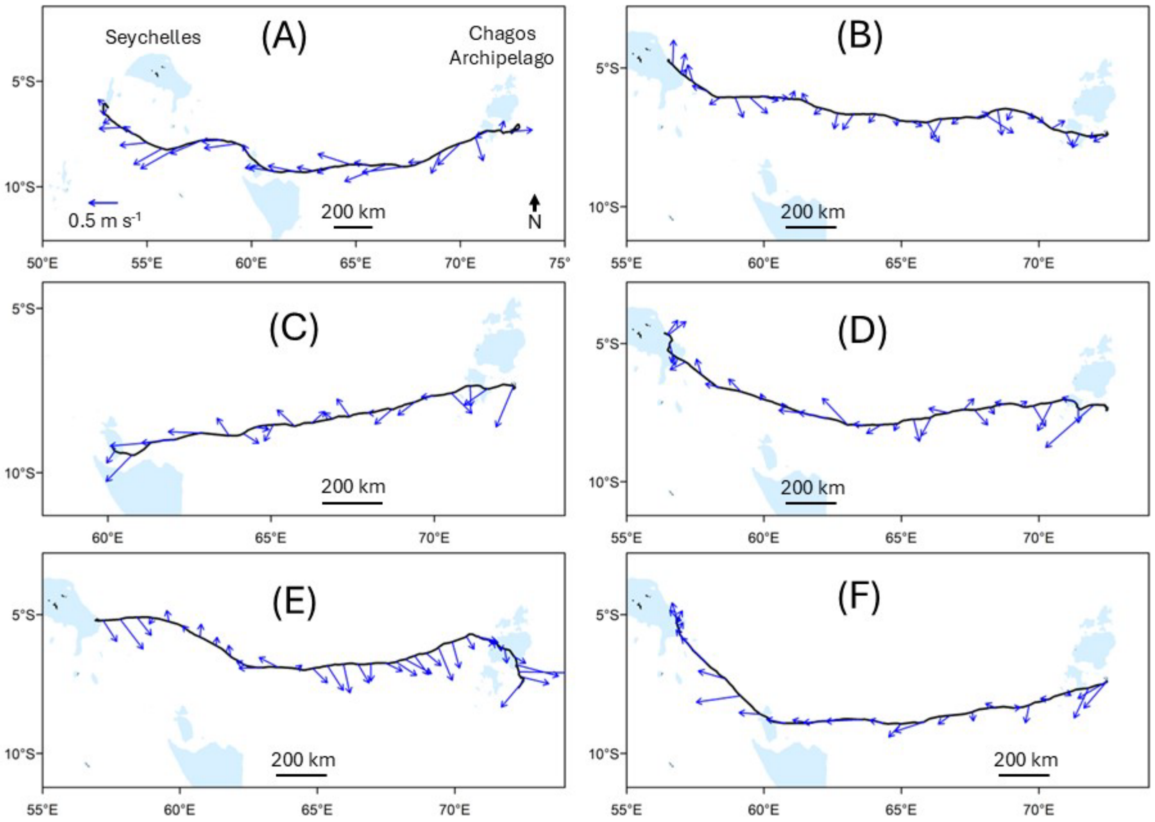

Fig. S5. Ocean currents associated with migration. The direction of ocean currents along each migration track. While the ocean current was determined for 1-h intervals, plotted for visual clarity are the mean current direction and speed per 24 h across each track. Panels A-F are turtles IDs #265002, #265004, #265006, #265007, #265008 and #265009. Direction of travel is from the Chagos Archipelago to the Seychelles in all cases.

Table S1. Fastloc GPS locations of satellite tracked green turtles. Times in UTC.

Table S2. Raw compass heading data (not corrected for declination) from satellite tracked green turtles. Times in UTC.
